# The moderating effects of mindful eating on the relationship between emotional functioning and eating styles in overweight and obese women

**DOI:** 10.1007/s40519-019-00740-6

**Published:** 2019-07-16

**Authors:** Kamila Czepczor-Bernat, Anna Brytek-Matera, Carla Gramaglia, Patrizia Zeppegno

**Affiliations:** 1 Katowice Faculty of Psychology, SWPS University of Social Sciences and Humanities, Technikow 9, 40-326 Katowice, Poland; 2 Dipartimento di Medicina Traslazionale, Psychiatry Institute, Università degli Studi del Piemonte Orientale, Via Solaroli 17, 28100 Novara, Italy; 3 Psychiatry Institute, Maggiore della Carità Hospital, C.so Mazzini 18, 28100 Novara, Italy

**Keywords:** Overweight, Obesity, Mindful eating, Emotional dysregulation, Negative emotions, Eating styles

## Abstract

**Purpose:**

The aim of the current study was to examine the moderating effect of mindful eating on the relationship between emotional functioning and eating styles in overweight and obese women.

**Methods:**

One hundred and eighty four overweight and obese adult women (BMI 30.12 ± 3.77 kg/m^2^) were assessed with the Difficulties in Emotion Regulation Scale, the Positive and Negative Affect Schedule, the Three Factor Eating Questionnaire and the Mindful Eating Scale.

**Results:**

Mindful eating significantly moderated several of the relationships between emotional functioning and eating styles. At all levels of mindful eating, emotion dysregulation and negative affect are both associated with greater emotional eating, but with stronger associations for high levels of mindful eating. For people low in mindful eating, both emotion dysregulation and negative affect are associated with lower restrictive eating, and neither of them are associated with uncontrolled eating. For people high in mindful eating, neither emotion dysregulation nor negative affect are associated with restrictive eating, and only negative affect is associated with greater uncontrolled eating.

**Conclusion:**

When mindful eating techniques are included as part of an intervention for overweight or obese individuals, it is even more important that those interventions should also include techniques to reduce emotion dysregulation and negative affect.

**Level of evidence:**

Level V, descriptive study.

## Introduction

According to epidemiological data, overweight and obesity-related problems are increasing worldwide, also because of the poor effectiveness of long-term treatment methods, with relevant consequences for the population’s health and healthcare costs [1–4]. New explanatory models for the onset and maintenance of overweight and obesity have been assessed in the recent literature [5–10], underscoring the role of high levels of emotional dysregulation, high levels of negative emotions [11, 12] and low levels of mindful eating [13]. While it is widely acknowledged that eating styles in overweight and obese individuals are more maladaptive than those of normal body weight ones [14–16], and include behaviours such as overeating, emotional eating, uncontrolled eating, it is still necessary to better clarify the relation among these variables.

Abnormal emotional functioning may involve difficulty in correctly identifying, interpreting, and coping with emotions [17–19] and a chronic experience of high levels of negative emotions [18]. Emotional dysregulation entails a poor ability to use emotions as an important source of information [17, 19, 20], and the consequent use of maladaptive coping patterns with negative emotions and stress [18], which may include regulation of emotional states by food intake (for instance: eating, overeating or restricting eating in response to emotions) [9, 21–23].

Emotional, uncontrolled and restrictive eating are problematic eating behaviours [24, 25], respectively, characterized by food intake independent of hunger and satiety feelings, but rather based on emotional stimuli [26–28]; overeating accompanied by a sense of lack of control [11, 29]; adoption of food restrictions aimed to control and manage body weight [30]. Overall, problematic eating behaviours lead to the loss of natural and intuitive eating and difficulties in discriminating various emotions from a state of hunger or satiety [24, 27, 31], which can be treated and restored with the aid of mindfulness techniques [26, 32–35].

The concept of mindful eating [25, 36] refers to eating in a conscious way, with the aim of satisfying body physical needs and their associated pleasure, focusing on bodily sensations associated with food intake (intuitive eating), rather than “being on automatic pilot” and using food intake to regulate emotions [25, 36, 37].

Previous reports about non-clinical samples describe that a mindfulness state is negatively associated with unhealthy eating styles (e.g., emotional and uncontrolled eating) [38–40], while it is positively related to healthy eating behaviours [41, 42]. Moreover, evidence suggests that deficits in emotion dysregulation [9, 23, 43] and a high level of negative emotions [8, 22] play a relevant role in the development and maintenance of obesity. Because of their impact on emotional regulation, emotional self-awareness, and adaptive coping with affect, interventions using mindfulness and mindful eating techniques proved to be effective in reducing binge eating episodes, depressive symptoms and emotional eating, as well as in enhancing intuitive eating [21, 25, 26, 44, 45]. The effectiveness of mindful eating interventions in obese patients has been supported as well [31, 35, 46]. Meta-analyses of mindfulness interventions offered to obese patients have supported their effectiveness on weight loss and decreasing maladaptive eating behaviours [47–52], albeit with different effect sizes for BMI (e.g., small [51], moderate [47]). Furthermore, a longer distance between pre-test and follow-up was associated with greater weight loss [52]. Katterman et al. [49] highlight that the long-term studies of mindful eating interventions should be done to assess effectiveness of mindfulness interventions in reducing BMI.

There are still gaps in understanding the relation among emotional functioning and dysregulation, eating styles and mindful eating in overweight and obese women. In addition, to our knowledge, no study has been conducted to assess mindful eating as a moderator between emotional functioning and eating styles.

Therefore, the aim of our study was to: (1) assess whether the relationship between emotional dysregulation and eating styles (emotional eating, uncontrolled eating, restrictive eating) was moderated by mindful eating, (2) evaluate whether the relationship between negative emotions and eating styles (emotional eating, uncontrolled eating and restrictive eating) was moderated by mindful eating.

Mindful eating was expected to moderate the relationship between emotion dysregulation/negative emotions and eating styles (emotional eating, restrictive eating and uncontrolled eating); in other words, high or medium mindfulness would mean a non-significant relationship between dysregulation/negative emotions and eating style.

## Materials and methods

### Participants

Three hundred patients were recruited from September 2017 to January 2018 through flyer advertisement at the Center for Eating Disorders in Grand Poland, and at the Psychodietetics and Eating Disorders Treatment Center in Katowice, which are both institutions dedicated to the treatment of overweight/obese and eating disorder patients. The flyers were distributed by staff and included contact information of the researcher.

Patients were screened according to inclusion and exclusion criteria to ensure the best possible homogeneity of the study sample. Inclusion criteria were the following: body mass index (BMI) ≥ 25 kg/m^2^; female gender. Patients were excluded if they were unwilling to take part in the study, and in case of current axis I disorder comorbidity [53]. Exclusion criteria such as gender and axis I comorbidity were adopted in the present study according to previous research which showed that women show higher levels of abnormal eating behaviours than men [e.g., 54] and that patients (BMI ≥ 25 kg/m^2^) with axis I comorbidity function worse than those without mental issues [e.g., eating disorders; 55].

A total of 151 women met the eligibility criteria and participated in the study. Patients were supplied no fee or reimbursement for their participation. Demographic and clinical characteristics are reported in Table 1.

**Table 1:** Descriptive features of the sample

	*M* (SD)	Min	Max
Demographic and clinical characteristics			
Body mass index (kg/m^2^)	30.12 (3.77)	25.22	51.20
Age (years)	35.73 (20.40)	18	64

	*N* (%)
Category of BMI	
Overweight	83 (54.97)
Obesity	68 (45.03)
Clinical characteristics	
Every day weighing	
Yes	16 (10.60)
No	135 (89.40)
Action for weight loss	
Yes	55 (36.42)
No	96 (63.58)
Methods of weight loss	
Dieting	29 (19.21)
Physical activity	23 (15.23)
Use of laxatives	0 (0)
Vomiting	1 (.66)
Starvation	2 (1.32)
Number of meals a day	
1	0 (0)
2	9 (5.96)
3	35 (23.18)
4	71 (47.02)
5	32 (21.20)
6 and more	4 (2.64)
Snacking in between meals	
Always	14 (9.27)
Often	43 (28.48)
Sometimes	67 (44.37)
Rarely	25 (16.56)
Never	2 (1.32)
Products snacking in between meals (multiple choice)	
Fruits	44 (29.14)
Vegetables	5 (3.31)
Sweets	67 (44.37)
Other	33 (21.85)

### Measures

Patients’ weight and height were measured and body mass index (BMI) was calculated.

Assessment included the following questionnaires: the Difficulties in Emotion Regulation Scale [17], the Positive and Negative Affect Schedule [56], the Three Factor Eating Questionnaire [57] and the Mindful Eating Scale [58].

#### The Difficulties in Emotion Regulation Scale

It measures the overall level of emotional dysregulation. It consists of 36 items, grouped in 6 subscales: non-acceptance of emotional responses, difficulty in engaging in a goal-directed behaviour, impulse control difficulties, lack of emotional awareness, limited access to emotion regulation strategies and lack of emotional clarity [17, 59]. Each item is rated on a 5-point Likert scale (from 1—“almost never” to 5—“almost always”): lower scores indicate higher adaptive emotion regulation.

The standard forward–backward translation procedure was used to create the Polish version of the questionnaire. In the present study, Cronbach’s alpha reliability coefficient for total scores was 0.95.

#### The Positive and Negative Affect Schedule

It is a 20-item self-report measure of “negative” and “positive” emotions [56, 60]. Each item is rated on a 5-point Likert scale (from 1—“slightly or not at all” to 5—“very strongly”). The higher the score is, the higher the negative (NA; e.g., afraid, worried) and positive (PA; e.g., active, zestful) emotions will be.

The Polish version of the questionnaire was used [61]. In the present study, Cronbach’s alpha reliability coefficient was 0.94 both for the negative and the positive dimensions. In this study, only the negative affect subscale was used.

#### The Three Factor Eating Questionnaire (TFEQ-R18) [57]

This is an 18-item scale used to assess eating styles, and contains three subscales: emotional, uncontrolled, and restrictive eating. Items 1–17 item are rated on a 4-point Likert scale (1–13: from 1—“definitely false” to 4—“definitely true”; 14: from 1—“only at meal times” to 4—“almost always”; 15: from 1—“almost never” to 4—“almost always”; 16: from 1—“unlikely” to 4—“very likely”; 17: from 1—“never” to 4—“at least once a week”), while item 18 is rated on an 8-point Likert scale (from 1—“eating whatever you want, whenever you want it” to 8—“constantly limiting food intake and never giving in”; responses 1 and 2 were recoded into 1; 3 and 4 into 2; 5 and 6 into 3; and 7 and 8 into 4 [15]. The higher the score is, the more maladaptive the eating styles are.

The Polish version of the questionnaire was used [15]. In the present study, Cronbach’s alpha reliability coefficient for the above-mentioned subscales was 0.88, 0.88 and 0.77.

#### The Mindful Eating Scale

The Mindful Eating Scale [58] measures overall mindfulness in the domain of eating behaviours. It includes 28 items and 6 subscales: acceptance, awareness, non-reactivity, routine, distractibility, unstructured [58]. Each item is rated on a 4-point Likert scale (from 1—“never” to 4—“usually”); higher scores indicate more mindful eating.

The standard forward–backward translation procedure was used to create the Polish version of the questionnaire. In the present study, Cronbach’s alpha reliability coefficient for total scores was 0.88.

### Statistical analysis

Statistical analysis was performed with the Statistical Package for Social Sciences (version 22.0). Univariate and multivariate logistic regressions were used to investigate predictors of mindful eating. MACRO “PROCESS” [62] with bootstrap *N* = 10,000 was used to analyse the moderating effects. Moderation, unlike mediation, gives the possibility to determine the level at which the relation between the variable *X* and *Y* depends on the level of the third variable (*M*). According to Hayes [62] “when the goal is to uncover the boundary conditions of an association between two variables, moderation analysis is used. An association between two variables *X* and *Y* is said to be moderated when its size or sign depends on a third variable or set of variables”.

All tables were created according to Hayes guidelines [62]. These tables are based on the statistical model of Hayes moderation [62].

## Results

### Descriptive statistics for variables

To summarize the obtained data, descriptive statistics (mean, standard deviation, minimum and maximum) for mindful eating, emotional dysregulation, negative affect and eating styles are reported in Table 2.

**Table 2:** Descriptive statistic for variables

Variable	*M*	SD	Minimum	Maximum
Mindful eating	72.45	11.14	47.00	98.00
Emotional dysregulation	94.97	27.18	48.00	159.00
Negative affect	21.31	9.08	10.00	46.00
Eating styles				
Emotional eating	8.43	3.01	3.00	12.00
Restrictive eating	17.76	4.95	7.00	28.00
Uncontrolled eating	21.58	6.64	10.00	36.00

### Moderation analysis

Regarding the relationship between emotional dysregulation and eating styles, mindful eating was a significant moderator for emotional eating, *R *= 0.53*, F* (3, 147) = 18.63, *p *= 0.0001, MSE = 6.72, *R*^*2*^-chng = 0.03, and restrictive eating, *R *= 0.41, *F* (3, 147) = 9.06, *p *= 0.04, MSE = 20.92, *R*^*2*^-chng = 0.02, but not for uncontrolled eating, *R *= 0.69, *F* (3, 147) = 43.99, *p *= 0.0001, MSE = 23.69, *R*^*2*^-chng = 0.001 (see Tables 3, 4).

**Table 3:** Regression analysis testing moderating effect of mindful eating

	Emotional eating		Restrictive eating		Uncontrolled eating	
	*b*	*t*	*b*	*t*	*b*	*t*
Mindful eating (ME)	− 0.19	− 2.75**	− 0.45	− 3.62***	− 0.35	− 2.63**
Emotion dysregulation (ED)	− 0.07	− 1.48	− 0.23	− 2.73**	0.04	0.46
Negative affect (NE)	− 0.31	− 2.39**	− 0.59	− 2.41*	− 0.46	− 1.89
ME × ED	0.002	2.49*	0.001	1.93*	− 0.0001	− 0.34
ME × NA	0.01	3.30**	0.01	2.10*	0.01	2.33**

*b*—unstandardized coefficients

*p < 0.05, **p < 0.01, ***p < 0.001

**Table 4:** Relationships with disordered eating styles at different levels of mindful eating

Level of mindful eating	Emotional eating		Restrictive eating		Uncontrolled eating	
	*b*	*t*	*b*	*t*	*b*	*t*
Relationships with emotion dysregulation						
Low (below 1 SD)	0.03	3.06**	− 0.09	− 4.26***	0.01	0.66
Medium (*M*)	0.05	4.87***	− 0.06	− 3.03**	0.01	0.44
High (above 1 SD)	0.07	4.77***	− 0.03	− 1.22	0.00	0.15
Relationships with negative affect						
Low (below 1 SD)	0.07	2.68**	− 0.13	− 2.57*	0.05	0.91
Medium (*M*)	0.14	5.41***	− 0.05	− 0.97	0.14	2.81*
High (above 1 SD)	0.21	5.40***	0.04	0.48	0.23	3.14**

*b—*unstandardized coefficient, 1 SD—low score (61.32), *M—*medium score (72.45), + 1 SD—high score (83.58)

*p < 0.05, **p < 0.01, ***p < 0.001

Further analysis showed a significant moderation effect of mindful eating for the relationship between negative emotions and emotional eating, *R *= 0.55, *F* (3, 147) = 21.22, *p *= 0.0001, MSE = 6.47, *R*^*2*^-chng = 0.05, restrictive eating, *R *= 0.33, *F* (3, 147) = 5.86, *p *= 0.001, MSE = 22.34, *R*^*2*^-chng = 0.03 and uncontrolled eating, *R *= 0.71, *F* (3, 147) = 49.96, *p *= 0.0001, MSE = 22.26, *R*^*2*^-chng = 0.02 (see Tables 3, 4).

Greater restrictive eating was associated with lower levels of both emotional dysregulation and negative affect, but only at lower levels of mindful eating. For participants high in mindful eating, neither emotional dysregulation nor negative affect predicted restrictive eating. Regarding emotional eating, greater scores were positively associated with greater degrees of both emotion dysregulation and negative affect for all levels of mindful eating, although these relationships were most pronounced for medium and high levels of mindful eating. Regarding uncontrolled eating, greater scores were positively associated with greater degrees of negative affect, but only for medium and high levels of mindful eating.

## Discussion

Mindful eating significantly moderated several of the relationships between emotional functioning and eating styles. At all levels of mindful eating, emotion dysregulation and negative affect are both associated with greater emotional eating, but with stronger associations for high levels of mindful eating. For people low in mindful eating, both emotion dysregulation and negative affect are associated with lower restrictive eating, and neither of them are associated with uncontrolled eating. For people high in mindful eating, neither emotion dysregulation nor negative affect are associated with restrictive eating, and only negative affect is associated with greater uncontrolled eating. Regarding clinical implications, the treatment of overweight and obese patients should focus on an increase in mindful eating while dealing with emotional functioning and restrictive eating. The effectiveness of mindful eating techniques for the treatment of bariatric surgery and non-bariatric obese patients has been supported [35, 63–66], while little is known about the importance of this approach in overweight individuals. Training in mindful eating (which reduces restrictive eating) strengthens a non-judgmental eating attitude and enhances the ability to properly address nutritional needs (which is associated with food intake based on body wisdom and natural physiological signals of hunger satiety) [13, 25, 36, 63]. This is an interesting and novel finding, as research on mindful eating typically describes this kind of intervention as helpful in reducing binge eating, overeating and emotional eating but not in restrictive eating.

However, our findings are not consistent with the literature about this topic [64], showing that (non-clinical) individuals with restrictive eating are characterized by high levels of emotional dysregulation. That research hypothesizes that restrictive eating is the most commonly used coping strategy for non-clinical individuals with poor emotion regulation who lack adaptive coping skills [64]. However, our findings would mean that the relationship between emotional functioning and restrictive eating are different among overweight and obese women (compared to a non-clinical sample), because in our sample, greater emotion dysregulation and higher negative affect are associated with lower restrictive eating. The possible reasoning for such opposite findings can be related to the Hemmingsson theory [6]. This author (based on previous research) assumes that people who in the future will be obese, from childhood are characterized by abnormal emotional functioning with which they try to cope by eating. Observing the trajectories of developing abnormal eating behaviours in this group, food intake became more and more non-restrictive and uncontrolled and these processes resulted in weight gain. Ultimately, these individuals lose emotional, cognitive and behavioural control over food intake and cannot introduce the dietary restrictions for weight reduction [6].

The analyses regarding the moderation effect of mindful eating in the relationship between emotional or uncontrolled eating and emotional dysregulation did not yield any suggestion about possible clinical implications. Similarly, this is presented in relation to negative emotions because of difficulties in interpretation of results. Although the model referring to emotional eating is significant, it is not possible to specify the level of mindful eating that is recommended to achieve with patients (at each level of mindful eating, the relation between emotional regulation or negative emotions and emotional eating is significant). Likewise, the same models for uncontrolled eating were difficult to interpret. The first model was not significant and the second one was unintelligible, because (a) negative emotions were not linked (directly and significantly) to uncontrolled eating and (b) a low level of mindful eating in the relationship between negative emotions and uncontrolled eating is insignificant.

Taking into consideration the low levels of mindful eating, both emotion dysregulation and negative affect are associated with less unhealthy restrictive eating. Likewise, mindful eating seems to be harmful with respect to the other two forms of unhealthy eating styles, such that at high levels of mindful eating, both emotion dysregulation and negative affect were associated with even greater degrees of both emotional eating and uncontrolled eating.

These results would suggest a paradoxical indication for a therapy to reduce the mindfulness level, nevertheless this indication is not coherent with mindful eating practice protocols [25, 34, 67–69], because they assume that a high level of mindful eating should not only lead to a reduction in restrictive eating (associated with avoiding “forbidden food”) but also a decrease in emotional and uncontrolled eating [13, 25]. The BASICS technique achieves a reduction in the emotional eating level through mini-meditations connected with mindful meal and body scanning for uncontrolled eating (B—breathe and belly check for hunger and satiety before you eat; A—assess your food; S—slow down; I—investigate your hunger throughout the meal, particularly halfway through; C—chew your food thoroughly; S—savour your food [13, pp. 46–67]). Perhaps, there are some variables that can moderate the effect of mindful eating on emotional eating and uncontrolled eating. Therefore, other explanations for non-specific or insignificant relationships described above should be sought. Perhaps, the role of mindful eating in the relationship between emotional functioning and emotional and uncontrolled eating is particularly relevant in bariatric surgery candidates [31, 35, 70]. Moreover, Marks model highlights body dissatisfaction as an important element in the relationship between emotional functioning and eating behaviours in obese populations. Body dissatisfaction may thus influence eating style and emotional functioning [8]. These theoretical assumptions should be empirically verified. Aside from using mindful eating techniques in an intervention for overweight or obese individuals, it is also important to include other techniques that can reduce emotion dysregulation and negative affect.

The findings regarding uncontrolled eating are not consistent with the literature about mindful eating. Moreover, results regarding the moderation effect of mindful eating in the relationship between emotional eating led to a paradox, where mindful eating seems to exacerbate the negative affects of independent variables on emotional eating behaviours. Identifying inconsistencies and paradoxical effects could be important in research, because discrepant findings could help researchers to update knowledge about a research topic, for example, through identifying new variables that could explain previous inconsistencies.

Briefly, consistent with the theoretical model of obesity described by Hemmingsson [6], Marks [homeostatic theory of obesity; 8] and Raman [clinical obesity maintenance model; 9], the treatment of overweight and obese people should entail techniques focused on the development of mindful eating skills aimed at improvement of emotional functioning. Furthermore, the current results [21, 71, 72] demonstrate that dialectical behaviour therapy (focusing, among others, on emotional functioning) increases the levels of intuitive eating and adaptive emotion regulation while decreasing the levels of binge eating disorder symptoms, emotional eating and psychological distress, and with Wnuk’s and Du’s [25] model, which describes that mindful eating techniques improve emotional functioning (acceptance and tolerance emotions). Moreover, other studies show that mindfulness and mindful eating are associated with food intake (significant and negative relationship with fat and sugar consumption) [73] and mindfulness and emotion regulation abilities can be useful in bariatric surgery candidate [70]. According to what described above, we created a complex psychological intervention (focusing on regulations of emotions, mindful eating and body image) whose effectiveness is currently being evaluated in overweight patients.

To address some of the limitations of the current study, the sample size should be increased, and men should be included as well. Moreover, further research is needed to better investigate the relationship between emotional functioning and uncontrolled eating. Moreover, subsequent studies analysing the relationship between these variables should be longitudinal or experimental. Another limitation is using BMI as an outcome measure of body composition. Subsequent research should use more objective measurements of body composition such as electrical bioimpedance to determine the proportion of body fat relative to lean tissue. In addition, important results may be derived from studies comparing participants with and without mental health disorders. The use of self-reported measures may be a further limitation.

Finally, since effect size was small in all models (interpretation based on *R*^2^-chng like squared semipartial correlation coefficient: 0.02—small effect, 0.15—medium effect, and 0.35—large effect), the current results should be treated with caution and further supported by research in this field.

## Conclusion

Our findings highlight the importance of mindful eating in the context of restrictive eating in the treatment of overweight and obese people. Aside from using mindful eating techniques in an intervention for overweight or obese individuals, it is also important to include other techniques that can reduce emotion dysregulation and negative affect.
